# The relationship between proactive coping and mindfulness: cross-cultural analysis

**DOI:** 10.1192/j.eurpsy.2022.614

**Published:** 2022-09-01

**Authors:** E. Belinskaya, M. Djuraeva, O. Tihomandritskaya, E. Dubovskaya

**Affiliations:** Lomonosov Moscow State University, Faculty Of Psychology, Moscow, Russian Federation

**Keywords:** coping strategies, proactive coping, Mindfulness

## Abstract

**Introduction:**

The inconsistency of the available empirical data on personal and situational predictors of effective coping allows us to make an assumption about the presence of mediating variables. Their search can be centered both on the inclusion of parameters of a higher socio-cognitive level in the analysis, and on the procedural characteristics of coping, one of which is proactivity in coping. The construct of mindfulness satisfies both of these requirements.

**Objectives:**

The purpose of this study was to identify cross-cultural differences in the relationship between proactive coping strategies and the level of mindfulness

**Methods:**

Five Facet Mindfulness Questionnaire (“eastern” awareness), Scale of Mindfulness (“western” awareness), Proactive Coping Invertory. The sample was N = 452 (residents of Russia and Uzbekistan, age 18-25)

**Results:**

For Russians and Uzbeks, the indicators of “western” awareness are associated with the attitude to potential difficulties as a source of positive experience and with reflection in case of their occurrence of possible behaviors, cognitive assessment of their own resources and prediction of results, as well as with such a proactivity strategy as the search for instrumental support. Intercultural differences were noted in terms of “eastern” awareness: for Uzbeks, they are associated with a proactive coping strategy, and for Russians – with reflexive coping.

**Conclusions:**

Mindfulness has the potential of meta-cognitive function in a situation of assessing possible life difficulties and choosing proactive coping strategies.

**Disclosure:**

No significant relationships.

